# Model-based exploration of hypokalemia in dairy cows

**DOI:** 10.1038/s41598-022-22596-0

**Published:** 2022-11-17

**Authors:** Julia Plöntzke, Mascha Berg, Rainald Ehrig, Sabine Leonhard-Marek, Kerstin Elisabeth Müller, Susanna Röblitz

**Affiliations:** 1 Zuse Institute Berlin, Takustr. 7, 14195 Berlin, Germany; 2 Library and Department of Physiology, University of Veterinary Medicine, 30559 Hannover, Germany; 3 Clinic for Ruminants, Veterinary Medicine, Freie Universität Berlin, 14163 Berlin, Germany; 4 Computational Biology Unit (CBU), Department of Informatics, University of Bergen, 5008 Bergen, Norway

**Keywords:** Computer modelling, Differential equations, Systems biology, Computational biology and bioinformatics, Computational models

## Abstract

Hypokalemia in dairy cows, which is characterized by too low serum potassium levels, is a severe mineral disorder that can be life threatening. In this paper, we explore different originating conditions of hypokalemia—reduced potassium intake, increased excretion, acid-base disturbances, and increased insulin—by using a dynamic mathematical model for potassium balance in non-lactating and lactating cows. The simulations confirm observations described in literature. They illustrate, for example, that changes in dietary intake or excretion highly effect intracellular potassium levels, whereas extracellular levels vary only slightly. Simulations also show that the higher the potassium content in the diet, the more potassium is excreted with urine. Application of the mathematical model assists in experimental planning and therefore contributes to the 3R strategy: reduction, refinement and replacement of animal experiments.

## Introduction

Potassium is a monovalent cation that is fundamental for cell functioning. It is involved in cell homeostasis and osmotic volume regulation^1,2^, in acid-base regulation^3^, formation of resting membrane potential, nerve impulse transmission, and muscle contraction. Its metabolism is a complex dynamical network. Potassium is taken up with feed, distributed between intra- and extracellular space, and excreted mainly with urine^4^. Milk contains about 1.4 g potassium per liter^5,6^ and is therefore an important contributor to excretion and loss from the organism, especially in high yielding dairy cows.

Potassium balance disorders can be life-threatening. Hypo- and hyperkalemia describe extracellular potassium below or above its physiological range of 3.9–5.2 mmol/L^7^, respectively. Hypokalemia is described frequently in cows presented to a veterinary clinic^6,8^. Clinical records include displaced abomasum with or without volvulus, mastitis, retained placenta, metritis and hepatic lipidosis. A serum potassium concentration below 2.5 mmol/L reflects severe hypokalemia, and most animals will be weak or recumbent. A serum potassium concentration of 2.5–3.5 mmol/L reflects moderate hypokalemia, and some cattle will be recumbent or appear weak with depressed gastrointestinal motility^3^.

Hypokalemia can result from increased loss, trans-cellular shift, or decreased intake of potassium. Increased loss may occur through the kidney, gastrointestinal tract, or milk. Hypokalaemia can also occur as a result of a redistributing shift of potassium from the extracellular space into cells^9^. Risk factors described for hypokalemia include high milk yield, reduced feed intake (e.g. due to illness), sweating in the hot season^10^, systemic or intramammary glucocorticoid treatment (e.g. isoflupredone acetate, dexamethasone^3^), treatment with multiple doses of dextrose and insulin^8,11^, and gastrointestinal absorption problems (e.g. due to enteritis or diarrhea).

The aim of our work is to showcase how a systems biology approach based on a kinetic model can be used to advance our understanding of the physiological mechanisms underlying hypokalemia. Kinetic models have already been useful in areas like dairy cattle nutrition^12^, metabolism^13^, and reproduction^14^. However, even though systems biology approaches have already been advocated for many years^15^, and despite the progress that has been made on the genomic level^16^, kinetic models in animal sciences are still scarce. One reason is that many animal scientists are simply not acquainted with this approach, so we want to raise awareness with our work. Another reason is that the data needed to parameterize these models are often limited in quality and quantity. Therefore, kinetic models are usually developed iteratively and incrementally, meaning that the model needs to be validated and maybe refined every time new data is gathered. This process becomes easier if a model is extensible and reusable, that is, if it has been designed to evolve and be used beyond its original purpose^17^. We hypothesize that our previously developed model for potassium balance in dairy cows^18^ satisfies this criterion by challenging it with a variety of experiments on potassium metabolism from the literature. These experiments include decreased potassium intake, increased excretion with urin or milk, changes in blood pH, a hyperglycaemic clamp for the illustration of elevated insulin, and dexamethasone administration. This model-based approach allows us to study perturbations of potassium homeostasis in silico and to identify knowledge gaps on which future research can be focused.

## Methods

### Model development and validation

**Figure 1:**
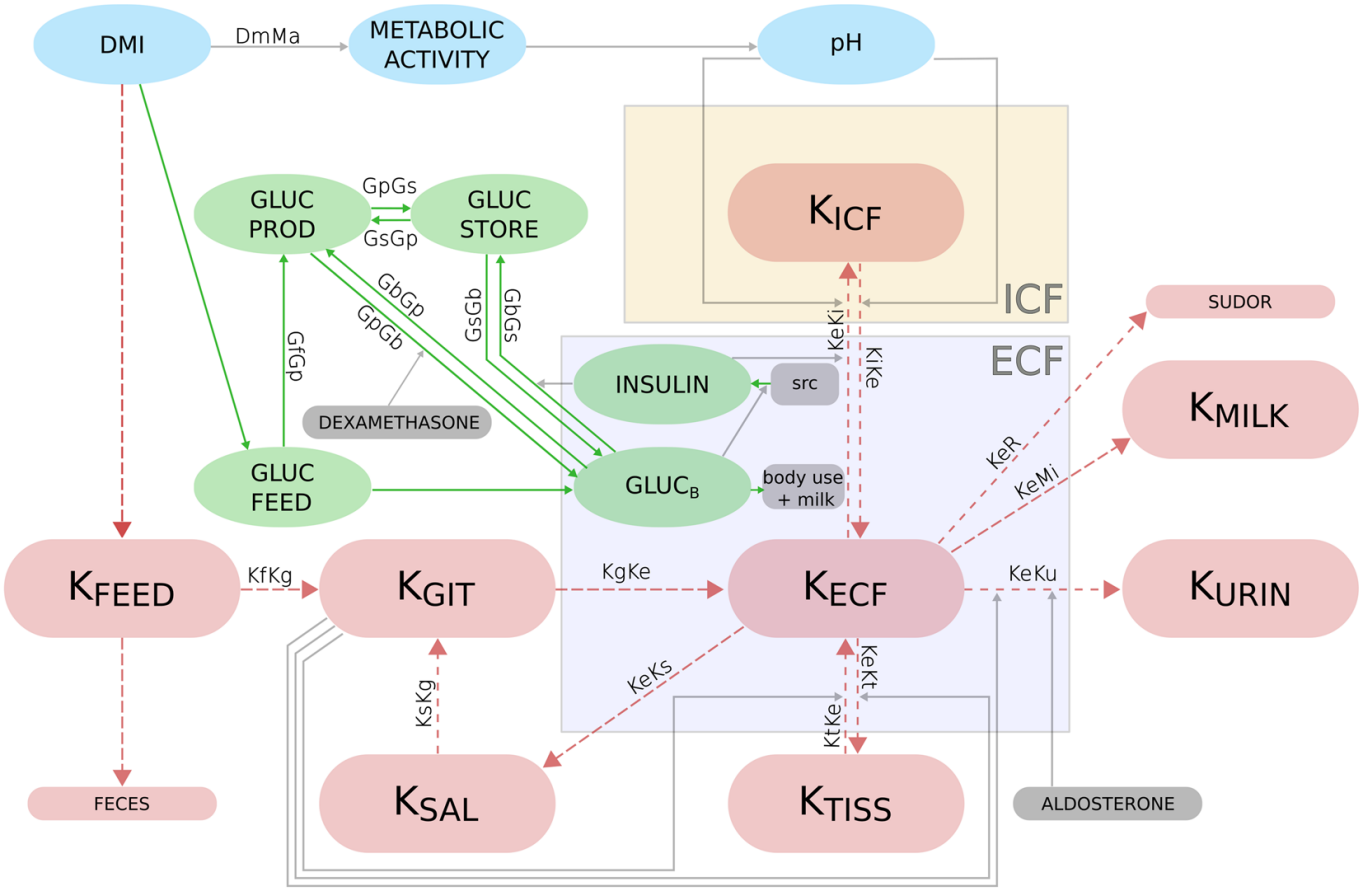
Schematic representation of the components and their relationships in the mechanistic model of potassium balance in cows. Each box represents a model component. The pink color represents potassium, green boxes correspond to metabolic components, and gray arrows and boxes indicate stimulatory or inhibitory effects. All components are fully explained in Table 1.

**Table 1:** Components and units in the model for potassium balance in dairy cows.

Component	Explanation	Unit	Initial value
DMI	Dry matter intake	g/h	487.5
$K_{FEED}$	Potassium taken up with the diet	g/h	5.543
$K_{ECF}$	Potassium in the extracellular blood fluid	g/L	0.187
$K_{ICF}$	Potassium in the intracellular blood fluid	g/L	0.937
$K_{URIN}$	Potassium excreted with urine, accumulated	g	0.0
$K_{GIT}$	Potassium in the gastrointestinal tract	g	30.872
$K_{TISS}$	Potassium in tissues except blood and bone	g	1509.6
$K_{SAL}$	Potassium in saliva	g	1.824
$K_{MILK}$	Potassium in milk, accumulated	g	0.0
*Insulin*	Insulin in blood	$\upmu$U/ml	22.287
$Gluc_{FEED}$	Glucogenic substances taken up with feed	g/h	146.25
$Gluc_{PROD}$	Pool of produced glucose	g	34.034
$Gluc_{B}$	Glucose in blood	g/L	0.556
$Gluc_{STOR}$	Glucose stored in tissues	g	3647.747
*Metabolic Activity (MA)*	Metabolic activity	–	6.249
*pH*	pH of blood	–	7.344

Note that the components $K_{ECF}$ and $K_{ICF}$ are calculated in g/L, whereas simulation results within this paper are shown in mmol/L.

**Table 2:** Rates and their units in the model for potassium balance in dairy cows.

Rate	Explanation	Unit
DmMa	Stimulation of metabolic activity by dry matter intake	–
GbGs	Glucose storage as glycogen in tissues (glycogenesis)	g/h
GfGb	Direct glucose absorption from the intestine to the blood	g/h
GfGp	Absorption of glucogenic substances	g/h
GpGb	Release of produced glucoseto the blood	g/h
GpGs	Storage of excess glucose in tissues	g/h
GsGb	Release of glucose from tissues to the blood	g/h
GsGp	Glucose production by glycogenolysis	g/h
KeKs	Potassum shift from extracellular blood fluid to saliva	g/h
KeKt	Potassium shift from extracellular blood fluid to other tissues	g/h
KeKu	Potassum excretion with urin	g/h
KeMi	Potassium excretion with milk	g/h
KeR	Potassium excretion with sweat	g/h
KeKi	Potassium shift from extracellular to intracellular blood fluid	g/h
KfKg	Amount of feed passing to the gastrointestinal tract	g/h
KgKe	Potassium absorption from the gastrointestinal tract to extracellular fluid	g/h
KiKe	Potassium shift from intracellular to extracellular blood fluid	g/h
KsKg	Potassium intake with saliva	g/h
KtKe	Potassium mobilisation from tissue into extracellular blood fluid	g/h
SnkGb	Metabolic use of blood glucose	g/h

The simulations were performed with an ordinary differential equation (ODE) model published by Berg et al.^18^, which can be used to simulate the potassium balance in non-lactating and lactating cows. The model had been designed on a whole organism level and includes mechanisms for dry matter intake, the glucose-insulin metabolism, potassium distribution between intra- and extracellular fluid, as well as potassium excretion, see Fig. 1. The model consists of two compartments (intra- and extracellular blood fluid), 12 ODEs, 4 algebraic equations, 63 model parameters, and experimental parameters. All model components and their units are listed in Table 1. The rates are explained in Table 2. The full equations and model parameters are listed in the Supplementary Information.

The model had been validated with data from literature and data from a clinical trial at Freie Universität Berlin. In this trial, potassium concentrations in blood were measured at several time points throughout a day in non-lactating cows without any treatment interventions^19^. The model can be downloaded as SBML file (Systems Biology Markup Language) from the BioModels database^20^ and can be run in, e.g., the CellDesigner software.

The starting point for all experiments is the default model setting^18,19^. It describes a non-lactating cow weighing 600 kg, fed 11.7 kg DMI per day with 1.137% potassium content. If changes are made in this setting, it will be stated in the description of the respective numerical experiment.

The dry matter intake (DMI) is modelled as a periodic function with period length of 24 h according to the day-night rhythm, whereby we assume that feed intake is higher at day time and lower during night. Therefore, all simulated components also show this periodic pattern.

### Model improvement

In the following, we describe the changes that were made in the model equations compared to the original model by Berg et al.^18^. We changed the parameter $p_{46}$, representing the fraction of glucose and glucogenic substances in the diet, from 0.08 to 0.3, since the latter value more accurately represents the carbohydrate fraction^21^. Furthermore, when performing simulations with very low to zero feed intake with the original model, we found that the renal potassium excretion was reduced to almost zero. However, literature describes an obligatory potassium excretion in vivo, which is a prerequisite for hypokalemia^9^. In cows, this potassium excretion is estimated to be 6 g in 24 h^21,22^. Hence, the parameter $p_{53}$, which represents the basic potassium excretion with urine in the rate *KeKu*, was changed from 0.01 to 0.25 g/h to meet the criterion of a basic potassium excretion of 6 g/day during fasting. Moreover, the linear dependency of the rate *KeKu* on $K_{ECF}$ was replaced by a threshold-dependent stimulation. This prevents *KeKu* from becoming too large, which would result in negative concentrations for $K_{ECF}$.

### Numerical experiments on hypokalemia

The numerical experiments were designed to simulate possible scenarios of hypokalemia. Hypokalemia in the model is characterized by a value of extracellular potassium, $K_{ECF}$, being less than 3.9 mmol/L^6^. Hypokalemia can result from whole body depletion, where the organism runs out of potassium, or from redistribution, where potassium is moved from extracellular into intracellular space. Glucose blood concentrations, represented by the model component $Gluc_B$, may also fall below their physiological range of 2.22–3.30 mmol/L (0.40–0.59 g/L)^7^. This state is referred to as hypoglycemia. Hypokalemia and hypoglycemia may lead to life-threatening conditions within the organism. If one of the two components, i.e., $K_{ECF}$ or $Gluc_B$, drops to zero, this marks the end of the numerical experiment.

#### Whole body depletion

Whole body depletion is characterized by too few potassium being available within the organism. Two scenarios are possible to arrive at this state: reduced potassium intake or increased potassium excretion. Decreased potassium intake over a long time period leads to hypokalemia because of the obligatory potassium loss by the kidney^9^. In the model, the potassium intake can be reduced by reducing total DMI or by reducing the potassium content in DMI, $K_{FEED}$.

In the adult cow, an increased potassium excretion happens via the kidney, with milk or with feces. We simulate these different scenarios of whole body depletion in experiments 1–4.

### Experiment 1: reduced dry mater intake

In experiment 1, we simulate a reduced potassium intake by reducing *DMI*, within the default model setting and without milk production. DMI is reduced to zero after 100 h by multiplying the original *DMI* equation (see Supplementary Information) with a negative Hill function, which switches off *DMI* at time $t=p_{100}$. The equation reads

1 $$\begin{aligned} y_{DMI}=p_{54}\cdot 487.5\cdot \left( 1-sin\left( \frac{\pi \cdot t}{12}\right) \right) \cdot H^-(t, p_{100};15), \end{aligned}$$

with $$p_{100}=100$$ h.

### Experiment 2: reduced potassium content in feed

In experiment 2, the potassium content in DMI, $K_{FEED}$, is varied. To achieve values for $K_{FEED}$ of 13 g, 40 g, 80 g and 133 g, the model is simulated with the parameter $p_{56}$ set to 0.001137, 0.003411, 0.006822, and 0.01137, respectively. One should note that low values of $K_{Feed}$ can easily be simulated in silico, but are difficult to prepare in vivo since ruminants‘ diet is generally rich in potassium^23^.

### Experiment 3: increased potassium excretion with urine

For this experiment, the rate for potassium excretion via urin, *KeKu*, is multiplied with the experimental parameter $p_{80}$, which is set to values between 1 and 4, leading to an up to fourfold increased rate *KeKu*:

$$KeKu=p_{80}\cdot \left( \left( 1+ p_{13}\cdot H^+(y_{K_{ECF}},p_{24};5)\right) \cdot p_6 \cdot y_{K_{GIT}} \cdot \left( 1 +p_{16} \cdot y_1 \cdot H^+(y_{K_{ECF}},p_{22};10)\right) +p_{53}\cdot H^+(y_{K_{ECF}},p_{89};2)\right)$$

All other components are as in the default model setting.

### Experiment 4: increased potassium excretion with milk

In vivo, the potassium content in milk varies between individuals and depends on the lactational state^24^. Potassium content in milk is fixed in the model to be 1.4 mmol/L^6^. Since the model does not account for varying potassium content in milk, we can only increase overall milk yield. This is achieved by varying parameter $p_{55}$ [liter milk/h] between 0.0 and 2.0, which corresponds to a milk production of 0–48 L/day. All other components are in the default model setting.

#### Redistribution

In some conditions, the potassium shift from extracellular to intracellular space increases and may result in hypokalemia. This can be caused by medications, hormonal dysregulation, or raised blood pH^9^. In experiments 5, 6, and 7, we explore different scenarios of redistribution.

### Experiment 5: acidosis, alkalosis

Changes in blood pH affect the potassium distribution between extra- and intracellular space. In alkalosis, i.e. blood pH over 7.45, potassium shifts into the cells, whereas in acidosis, i.e. blood pH below 7.35^25^, potassium is shifted out of the cells. To change blood pH in the model, we introduce the experimental parameter $p_{85}$ into the equation for pH,

$$\begin{aligned} y_{pH} = p_{85}-\frac{y_{MA}}{40}. \end{aligned}$$

The model parameter is set to $p_{85}=\{7.2,\,7.35,\,7.5,\,7.65,\,7.8\}$, respectively, whereby $p_{85} = 7.5$ corresponds to the default model setting.

### Experiment 6: increased insulin

Insulin causes a dose related decline in extracellular potassium^26^, while potassium shifts into the intracellular space. We simulate the hyperglycaemic clamp as described in^26^ , where men are infused with glucose solution until reaching a steady state in blood glucose. Responsively, the adrenal glands increase excretion of insulin in order to decrease the blood glucose. During the experiment, the steady state of blood glucose is maintained by further infusions of the same. In the model, we manually set the component $Gluc_B$ to 1.2 g/L at $t = 150$ h for the next 50 h.

### Experiment 7: dexamethasone administration

Dexamethasone treatment is frequently applied in cows with ketosis to increase available glucose in the blood serum^27,28^. Furthermore, dexamethasone treatment is described together with decreasing serum potassium concentrations in dairy cows^11,29^ and in goats^30^. Complications of dexamethasone treatment can be wide spread. Kojouri et al.^31^, for example, report a case of atrial fibrillation after ketosis treatment in conjunction with hypokalemia.

We implemented dexamethasone administration in the model to explore its interaction with the glucose-insulin and potassium dynamics, thereby following the dosing scheme from an in vivo experiment at the clinic for Ruminants, Freie Universität Berlin^19^. Non-lactating cows were treated with Voren Suspension (Dexamethason-21-isonicotinat), using a dose of 0.02 mg/kg body weight as recommended by the producing company Boehringer Ingelheim, which results in a dose of 12 mg for a 600 kg cow, the default body weight in the model.

The pharmakokinetics of Dexamethason-21-isonicotinat was described by Toutin et al.^32^. In their study, the dose was 0.1 mg/kg body weight, as recommended by the producer at that time, and the pharmacokinetic parameters were determined to be $t_{max} = 3.6$ h, $c_{max}= 44.1$ ng/ml and $AUC = 646.2$. Based on the approach described in Stötzel et al.^33^, the change of dexamethasone concentration in the blood (in ng/ml) is calculated in our model as

$$\begin{aligned} \frac{d}{dt}y_{Dexa}=D\cdot \beta ^{2} \cdot t\cdot e^{-\beta \cdot t} - c_{Dexa} \cdot y_{Dexa}. \end{aligned}$$

The parameter *D* represents the amount of administered drug, $\beta$ is an inverse scale parameter that determines the shape of the area under the curve, and $c_{Dexa}$ denotes the clearance rate of $y_{Dexa}$. The values of these three parameters were computed from the pharmacokinetic parameters in^32^ such that the simulated concentration profile of dexamethason agrees with the dynamics described therein:

$$\begin{aligned} D=11.8 \text { ng/ml},\quad \beta =1.1418 /\text {h},\quad c_{Dexa}=0.106 /\text {h} \end{aligned}$$

Today, a fivefold smaller dose of 0.02 mg per kg bodyweight is recommended by the producer compared with the dose applied in^32^. To account for this fact, we scaled the parameter *D* accordingly to $D=2.36$ ng/mL.

Dexamethasone administration in the model stimulates the rate *GpGb*, i.e., the release of glucose produced by the liver via gluconeogenesis to the circulating blood. The rate is therefore modified as follows:

$$\begin{aligned} GpGb \cdot (1 + y_{Dexa} \cdot p_{74}), \quad p_{74} = 3 \end{aligned}$$

This represents the stimulating effect of dexamethasone on gluconeogenesis observed in vivo^27,34^.

## Results and discussion

### Whole body depletion

In the model, reduced potassium intake with *DMI* or $K_{FEED}$ leads to reduced extracellular potassium concentrations, $K_{ECF}$. As a consequence, the amount of potassium excreted with urine, $K_{URIN}$, is also reduced because the rate of urinary potassium excretion, *KeKu*, depends on $K_{GIT}$ and $K_{ECF}$ (compare the full notation of equations and rates in the Supplementary Information). The obligatory urinary potassium loss finally leads to hypokalemia and depletion of extracellular and intracellular potassium, as well as depletion of the store, $K_{TISS}$.

In vivo, magnesium depletion is described to aggravate severe hypokalemia by increasing renal potassium loss^35^. Increased excretion via the kidney may be caused by diuretic treatment or aldosterone increase. Aldosterone may increase via the renin-angiotensin-aldosterone-system, e.g., if the blood volume decreases. Increased aldosterone may cause metabolic alkalosis^9^, which can worsen the diuretic-induced hypokalemia by increasing the potassium shift into cells. Diarrhea can also cause increased potassium loss. It is frequently observed in calves, but also adult cows can suffer from diarrhea, following indigestion with energetic and metabolic imbalances.

#### Experiment 1: reduced dry matter intake

**Figure 2:**
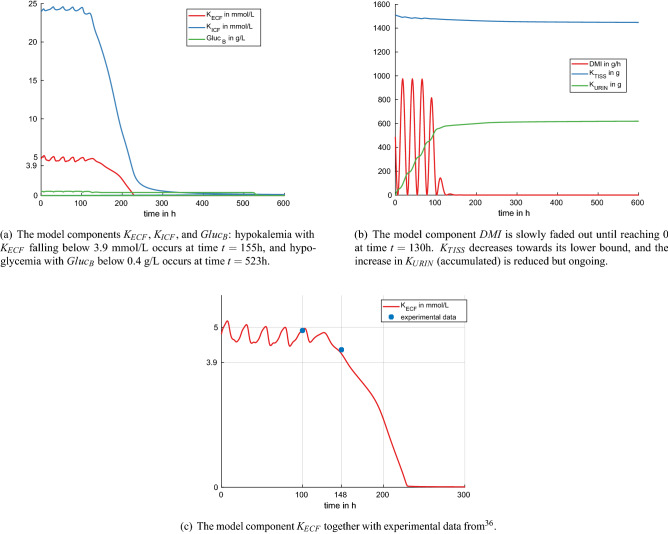
Experiment 1: reduced potassium intake by reducing DMI to zero after 100 h (no milk production, default model setting).

The results of reducing DMI to 0 g/h after 100 h are presented in Fig. 2. Figure 2a shows the components $K_{ECF}$ and $Gluc_B$. Hypokalemia occurs at time $t = 155$ h, hypoglycemia at time $t = 523$ h. Figure 2b shows that $K_{TISS}$ decreases towards its lower bound of about 1444 g, and that the urinary excretion with $K_{URIN}$ is reduced but ongoing. In Fig. 2c, experimental data from Clabough and Swanson^36^ are presented together with the simulation results of $K_{ECF}$. Clabough et al.^36^ studied six non-pregnant, non-lactating Holstein cows, fasted for 48 h. They measured mean serum potassium values before and after 48 h of fasting being 4.9 and 4.3 mmol/L. The simulated dynamics in experiment 1 and the experimental data from Clabough and Swanson^36^ match very well. In another study, plasma potassium in beef steers fasted for up to four days fell from 4.1 to 3.7 mmol/L^37^. Compared to the simulation results, the relatively smaller decline in steers over a longer fasting period may be due to the fact that steers have more muscle mass and hence more potassium storage capacity.

#### Experiment 2: reduced potassium content in feed

**Figure 3:**
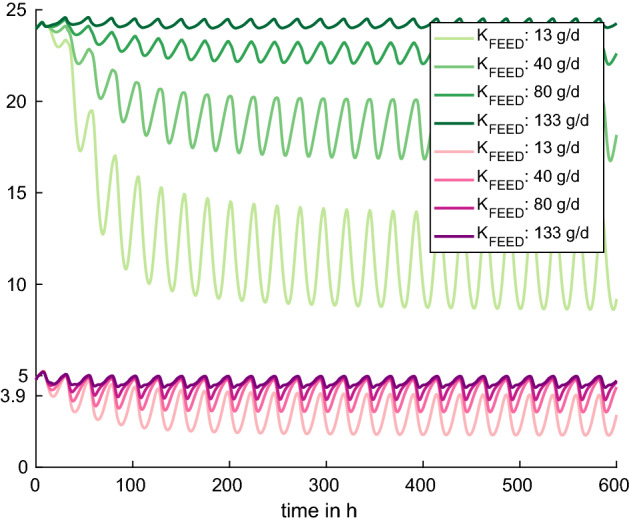
Experiment 2: We observe reduced potassium intake by decreasing $K_{FEED}$ from its default value of 133 g/day to 13 g/day. With decreasing $K_{FEED}$, $K_{ECF}$ (pink, in mmol/L) and $K_{ICF}$ (green, in mmol/L) decrease as well. For the lowest experimental value $K_{FEED}=13$ g/day, hypokalemia occurs after 35 h.

In Fig. 3a, $K_{FEED}$ is lowered from its default value of 133 g/d to 13 g/day. With decreasing potassium intake, $K_{ECF}$ and $K_{ICF}$ are decreasing as well. However, as it is visible from the simulations, extracellular potassium levels decrease only slightly, whereas the decrease in intracellular potassium is much more pronounced, which nicely illustrates the buffering function. For $K_{FEED}=13$ g/day, hypokalemia occurs after 35 h.

Since ruminants’ diet is generally rich in potassium, a deficient diet in vivo is challenging to prepare^23^. Nevertheless, Pradhan and Hemken^23^ managed to perform experiments with potassium deficient diets in lactating dairy cows. In their study, diets with a potassium content of 0.06% and 0.15% were fed to lactating cows. Clinical symptoms such as floor licking, changes in skin and hair appeared after 3–4 weeks of deficient diet. Unfortunately, the authors only state one mean experimental measurement of serum potassium per cow for the studied period. The mean serum potassium values range from 3.5 mmol/L to 5.1 mmol/L in the 0.06% diet group, and from 4.5 mmol/L to 6.9 mmol/L in the 0.15% diet group. For comparison with experiment 2, we calculated the potassium content in DMI on the basis of the default DMI (11.7 g) to be 7 g and 17 g for the 0.06% and 0.15% diet group, respectively. In the simulations with $K_{FEED}=13$ g/day and 40 g/day, the mean values of $K_{ECF}$ over 300 hours were calculated to be 3.24 mmol/L and 4.13 mmol/L, respectively, the minimum values are 1.79 mmol/L and 3.01 mmol/L. The results, however, are difficult to compare since most of the stated serum potassium values in Pradhan and Hemken^23^ are above the hypokalemic threshold even though clinical symptoms are reported. This can possibly be attributed to an imprecise potassium assay technique back then and the fact that only one mean value for the whole study period is reported.

#### Experiment 3: increased potassium excretion with urine

**Figure 4:**
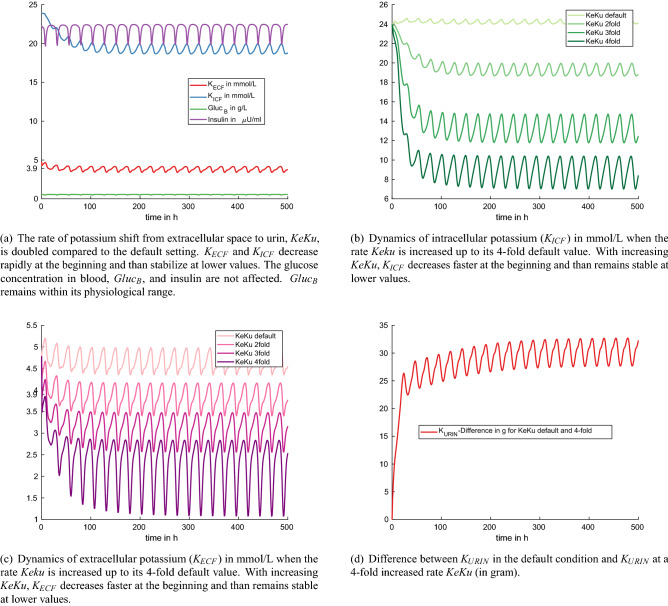
Experiment 3: increased potassium excretion with urine by increasing the rate *KeKu* up to the fourfold of its default setting.

Figure 4a shows the model dynamics resulting from a twofold increase of the rate for urinary potassium excretion, *KeKu*. We observe that $K_{ICF}$ and $K_{ECF}$ are initially decreasing rapidly and then stabilize at lower values. Hypokalemic values for $K_{ECF}$ are reached within 17 h after the start of the experiment. Glucose in the blood and insulin stay within their physiological ranges. Figures 4,b,c show the dynamics of $K_{ICF}$ and $K_{ECF}$ when the rate *KeKu* is increased up to the fourfold of its default value. With an increasing rate *KeKu*, $K_{ECF}$ and $K_{ICF}$ decrease faster at the beginning and then remain stable at lower values throughout the simulation period. Figure 4d shows the difference between $K_{URIN}$ in the default condition and $K_{URIN}$ at the fourfold increased rate *KeKu*. This difference rapidly increases within the first 24 h and then starts oscillating between 27 and 33 g. The trend in the predicted urinary excretion with potassium agrees with the observation in St. Omer and Roberts^22^: the higher the potassium content in the diet, the more potassium is excreted with urine. The renal excretion of potassium with $K_{URIN}$ in the model is not related to other minerals or acid base components, as it is crucial in vivo. Hence, the numerical experiments explore the quantitative dynamics solely inside the potassium network. Simulations mainly predict that increased urinary potassium excretion with $K_{URIN}$ finally leads to depletion of $K_{ECF}, K_{ICF}$, and $K_{TISS}$.

#### Experiment 4: increased potassium excretion with milk

**Figure 5:**
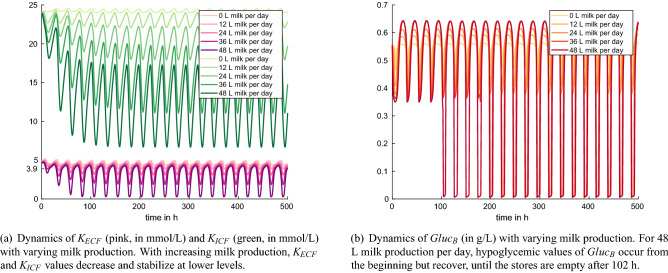
Experiment 4: increased potassium excretion with milk (default model setting, milk yield variation between 0 and 48 L milk per day).

With increasing milk production, the values for intra- and extracellular potassium content, $K_{ECF}$ and $K_{ICF}$, decrease and stabilize at lower values (Fig. 5a). The blood glucose, $Gluc_B$, is maintained within its physiological limits until stores are empty (Fig. 5b). For 48 L milk production per day, hypokalemic values of $K_{ECF}$ can be observed after 9 h. Values of $Gluc_B$ are hypoglycemic right from the beginning but recover until the stores are empty after 102 h. Since the model does not allow for varying potassium content in milk, milk increase leads to early hypokalemia.

### Redistribution

Changes in blood pH or medications can increase the shift of potassium from extracellular to intracellular space. The results of the corresponding numerical experiments are discussed in the following.

#### Experiment 5: acidosis, alkalosis

**Figure 6:**
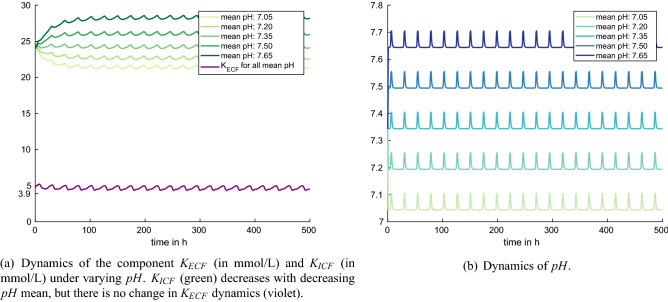
Experiment 5: variations in the mean *pH* value (default model setting).

Figure 6 shows the dynamics of the model components $K_{ECF}$, $K_{ICF}$, and *pH* during the experiments. When the model parameter $p_{85}$ is set to $p_{85}=\{7.2,\,7.35,\,7.5,\,7.65,\,7.8\}$, this leads to mean pH values of 7.05, 7.2, 7.35, 7.5, and 7.65, respectively. With increasing *pH*, also $K_{ICF}$ increases, whereas the acid-base variations in the numerical experiments do not have any effect on extracellular potassium levels $K_{ECF}$ (Fig. 6a). The latter is in contradiction to experimental findings in humans^9^, and hence probably also in contradiction to what is happening in cows. Rastergar and Soleimani^9^ describe the behaviour of potassium under changing pH conditions in humans. They observed that for each 0.1 unit change in pH, there is a change of approximately 0.6 mmol/L serum potassium, varying greatly by the nature of the acid-base disorder. In a recent study of cows with left displaced abomasum, Ismael et al.^38^ observed alkalic blood pH values and hypokalemic potassium values (mean values in affected cows). It seems that the mechanisms describing the acid-base metabolism in the model are not sophisticated enough to have an effect on $K_{ECF}$. More precisely, *pH* influences the rate *KeKi* more than the rate *KiKe*. In addition, the effect parameter $p_{21}$ is too small to have an appropriate effect on the rate *KiKe*.

#### Experiment 6: increased insulin

**Figure 7:**
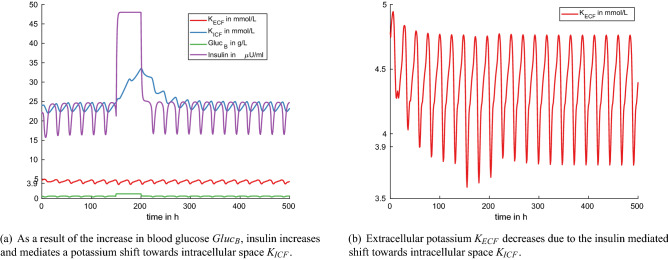
Experiment 6: hyperglycaemic clamp with the model component $Gluc_B$ set manually to 1.2 at time $t=150$ h for 50 h (default model setting with $p_{55}=0.9$, $p_{54}=1.2$).

In the simulated hyperglycemic clamp the model component $Gluc_B$ is manually set to 1.2 at time $t=150$ h for a duration of 50 h. As a consequenc, insulin increases and also the rate of potassium shift from extra- to intracellular space, *KeKi*. Hence, we observe an increase in the component $K_{ICF}$ (Fig. 7a) and a decrease in $K_{ECF}$ (Fig. 7b). All components return to their previous levels after the end of the experiment without further intervention.

We compare the simulation results with measurements from Grossen-Rösti et al.^39^, who studied hyperglycemic clamp dynamics in 11 dairy cows at different times around parturition. The authors report a steady state glucose concentration between 1.07 and 1.12 g/L, which is comparable with the simulated steady state of $Gluc_B = 1.2$ g/L in our model. In the study, insulin was measured before the beginning of manipulation as less than 20 $\upmu$U/mL. Insulin rose up to 140 $\upmu$U/mL and 78 $\upmu$U/mL in non-lactating pregnant cows and in postpartum lactating cows, respectively, while glucose was in the steady state clamp condition. In the simulation, insulin increases to a maximum of 48 $\upmu$U/mL. This means that the insulin increase due to blood glucose increase is less pronounced in the model compared to the experimental measurements from Grossen-Rösti et al.^39^.

The authors in DeFronzo et al.^26^ studied potassium shift due to a HGC in 29 humans. In the HGC condition the mean serum potassium decreased by $0.54\pm 0.04$ mmol/L. In the model simulation, the 24 h mean value of $K_{ECF}$ before the start of the experiment was 4.36 mmol/L, and it decreased to a 24 h mean of 4.31 mmol/L during HGC, with a minimal value of 3.59 mmol/L. This average decrease of 0.05 mmol/L during HGC is very small compared to the study^26^. This can be attributed to the oscillatory dynamic behavior of potassium in the model. The difference in serum potassium between feed intake and fasting is increased during HGC, while maximum peak concentrations decrease only slightly.

#### Experiment 7: dexamethasone administration

**Figure 8:**
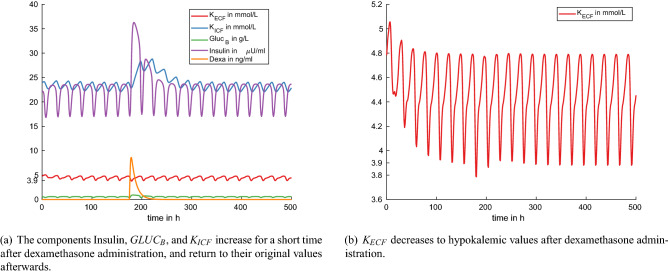
Experiment 7: dexamethasone administration at time $t = 176$ h (default model setting with $p_{55} = 1$ L/h, i.e. 24 L milk production per day).

Upon dexamethasone administration, the glucose level in blood, $Gluc_{B}$, rises, followed by a rise in insulin (Fig. 8a). Insulin enhances the rate for potassium transport from extra- to intracellular space, *KeKi*. This potassium shift to the intracellular space is visible in the simulation result as an increase in $K_{ICF}$ (Fig. 8a). Coffer et al.^29^ studied the effect of a single dose of 20 mg dexamethasone in seven cows between days 20–25 of lactation. They report the mean values of blood glucose and serum potassium in their Figs. 1 and 3, from where we extracted the following values for comparison with our simulation results. Glucose blood concentrations increased from an average of 0.48 g/L (mean of the seven studied cows) before dexamethasone administration by 0.44 g/L up to a maximum value of 0.92 g/L at 24 h after drug administration. Note, however, that the authors measured only every 24 h such that the peak concentration could have occurred at any time between $t=0$ and $t=48$ h. In the model simulations, the maximum value $Gluc_B=0.91$ g/L is reached seven hours after drug administration (Fig. 8a). Compared to the value $Gluc_B=0.59$ g/L attained 24 h before administration, this corresponds to an increase of 0.32 g/L in the model. In the study by Coffer et al.^29^, serum potassium levels increased very little after dexamethasone administration and decreased towards the pre-treatment value within 24 h after treatment. In the simulation, we observe a very small decrease in extracellular potassium down to a minimum of $K_{ECF}=3.8$ mmol/L four hours after drug administration (Fig. 8b). This decrease is caused by the increase in blood glucose, $Gluc_B$, which causes an increase in insulin and hence an increase in the rate of potassium shift from extra- to intracellular space, *KeKi*. This difference in qualitative behavior could hint to the fact that the effect parameter $p_{74}$ needs to be adapted in a more sophisticated manner. For this purpose, however, time series data would be needed.

On the other hand, there is good quantitative agreement with a measurement value reported in Kojouri et al.^31^. In this case study about atrial fibrillation and hypokalemia, the authors report a serum potassium value of 3.5 mmol/L after dexamethasone administration in a postpartum cow.

## Conclusion and outlook

We have illustrated that the model for potassium balance in dairy cows can be used to simulate the dynamics of hypokalemia for a number of scenarios described in literature. In most cases, model simulations agree qualitatively, in some cases even quantitatively, with clinical studies. To obtain these results, only minor modifications needed to be made on the original model. This demonstrates the capability of our model to evolve and be used beyond its original purpose, i.e., its extensibility and reusability.

We have also identified some mismatches between model simulations and results from literature. Some of these mismatches can be attributed to imprecise data reported in literature, e.g. imprecise potassium assay techniques or lack of information about measurement time w.r.t. last feed intake. The latter is important because there are fluctuations in potassium levels depending on the time of feed intake, which is also visible in our model simulations. In addition, the definition of hypokalemia is inconsistent. The feasibility of the threshold $K_{ECF}=3.9$ mmol/L cannot be evaluated with our simulations because the numerical experiments neither included treatment nor correlated symptoms with plasma values of potassium. Other mismatches between model simulations and data are due to insufficiencies in the potassium balance model, for example in the mechanisms describing the acid-base metabolism. Further adjustments of model equations and parameters based on experimental time-series data are needed to make the model even more reliable in future.

A previous review on hypokalemia in cattle^3^ points out the importance of considering extra- and intracellular potassium dynamically to evaluate more precisely the whole body situation with respect to clinical assessment and prognosis of the patient. Hence, we believe that further research on this topic can benefit from a model-based approach. For example, experiments on hyperkalemia and the treatments of hypo- and hyperkalemia could be simulated in silico. Furthermore, one could include sodium in order to study more precisely the intra- and extracellular shifts and renal excretion mechanisms.

Finally,the model may be used in teaching and research situations in which knowledge of potassium (or other nutrient) metabolism is important, but in which there is truly no need to go to the expense of repetitive animal experimentation. We hope that this type of modeling will make it possible to test new therapeutic approaches in silico first, thus filtering out the most promising approaches before carrying out experiments on animals. In that way, modeling and simulation are useful tools for the reduction, refinement and replacement of animal experiments^40,41^.

## Supplementary Information

Supplementary Information.

## Data Availability

All data generated or analysed during this study are included in this published article and its supplementary information files.

## Code availibility

Accession codes: The code for the updated model is available from the BioModels database: https://www.ebi.ac.uk/biomodels/MODEL2201250001. The software to run the model can be downloaded from http://www.celldesigner.org
