# Supplementary material for: Model-based exploration of hypokalemia in dairy cows
Source: Sci Rep. 2022 Nov 17;12:19781. doi: 10.1038/s41598-022-22596-0 (PMC9672062; doi:10.1038/s41598-022-22596-0)
Supplement: Supplementary file 1 — Supplementary Information. [file 41598_2022_22596_MOESM1_ESM.pdf]

Model-based exploration of hypokalemia in dairy cows: Supplementary Information

Julia Plöntzke[1,*], Mascha Berg[1], Rainald Ehrig[1], Sabine Leonhard-Marek[2], Kerstin Elisabeth Müller[3], and Susanna Röblitz[4]

[1]Zuse Institute Berlin, Takustr. 7, 14195 Berlin, Germany
[2]Library and Department of Physiology, University of Veterinary Medicine, 30559 Hannover, Germany
[3]Clinic for Ruminants, Veterinary Medicine, Freie Universität Berlin, 14163 Berlin, Germany
[4]Computational Biology Unit (CBU), Department of Informatics, University of Bergen, N-5008 Bergen, Norway
[*]corresponding author: ploentzke@zib.de

ABSTRACT

Hypokalemia in dairy cows, which is characterized by too low serum potassium levels, is a severe mineral disorder that can be life threatening. In this paper, we explore different originating conditions of hypokalemia – reduced potassium intake, increased excretion, acid-base disturbances, and increased insulin – by using a dynamic mathematical model for potassium balance in non-lactating and lactating cows. The simulations confirm observations described in literature. They illustrate, for example, that changes in dietary intake or excretion highly effect intracellular potassium levels, whereas extracellular levels vary only slightly. Simulations also show that the higher the potassium content in the diet, the more potassium is excreted with urine. Application of the mathematical model assists in experimental planning and therefore contributes to the 3R strategy: reduction, refinement and replacement of animal experiments.

Model equations

$$
\begin{aligned}
y_{DMI} &= p_{54} \cdot 487.5 \cdot \left(1 - sin\left(\frac{\pi \cdot t}{12}\right)\right) \\
\frac{d}{dt}(y_{Gluc_B} \cdot V_{extra}) &= GfGb - GbGs + GsGb - SnkGb + GpGb \\
y_{Gluc_{FEED}} &= p_{46} \cdot y_{DMI} \\
\frac{d}{dt} y_{Gluc_{PROD}} &= GfGp - GpGb - GpGs + GsGp \\
\frac{d}{dt} y_{Gluc_{STOR}} &= GbGs - GsGb + GpGs - GsGp \\
\frac{d}{dt}(y_{Ins} \cdot V_{extra}) &= p_{49} \cdot y_{Gluc_B} - p_{41} \cdot y_{Ins} \\
\frac{d}{dt}(y_{K_{ECF}} \cdot V_{extra}) &= KgKe - KeKu - KeKt + KtKe + KiKe - KeKi - KeR - KeKs - KeKm \\
y_{K_{FEED}} &= p_{56} \cdot y_{DMI} \\
\frac{d}{dt} y_{K_{GIT}} &= KfKg - KgKe + KsKg \\
\frac{d}{dt}(y_{K_{ICF}} \cdot V_{intra}) &= KeKi - KiKe \\
\frac{d}{dt} y_{K_{MILK}} &= KeKm \\
\frac{d}{dt} y_{K_{SAL}} &= KeKs - KsKg \\
\frac{d}{dt} y_{K_{TISS}} &= KeKt - KtKe \\
\frac{d}{dt} y_{K_{URIN}} &= KeKu \\
\frac{d}{dt} y_{MA} &= DmMa - p_{44} \cdot y_{MA} \\
y_{pH} &= 7.5 - \frac{y_{MA}}{40}
\end{aligned}
$$

Rates

$$\begin{aligned}
DmMa &= p_{45}+p_{42}\cdot H^{+}(y_{DMI},p_{43};2)\\
GbGs &= H^{+}(y_{Gluc_{FEED}},p_{1};10)\cdot p_{12}\cdot y_{Gluc_{PROD}}\cdot H^{-}(y_{Gluc_{STOR}},p_{57};10)\cdot y_{Ins}\cdot y_{Gluc_{B}}\cdot H^{-}(p_{55},p_{36};10)\\
GfGb &= p_{48}\cdot y_{Gluc_{FEED}}\\
GfGp &= (1-p_{48})\cdot y_{Gluc_{FEED}}\\
GpGb &= p_{37}+p_{39}\cdot y_{Gluc_{PROD}}\cdot H^{-}(y_{Gluc_{B}},p_{50};10)\\
GpGs &= p_{51}\cdot y_{Gluc_{PROD}}\cdot H^{-}(y_{Gluc_{STOR}},p_{57};10)\cdot H^{-}(p_{55},p_{62};10)\\
GsGb &= H^{-}(y_{Gluc_{FEED}},p_{1};10)\cdot p_{17}\cdot (p_{7}-y_{Gluc_{B}})\cdot H^{+}(y_{Gluc_{STOR}},p_{35};10)\\
&\quad +H^{-}(p_{55},p_{64};10)\cdot H^{-}(y_{Gluc_{B}},p_{65};10)\cdot p_{63}\cdot H^{+}(y_{Gluc_{STOR}},p_{35};10)\\
GsGp &= p_{60}\cdot H^{-}(y_{K_{FEED}},p_{61};5)\cdot H^{+}(y_{Gluc_{STOR}},p_{35};10)\\
KeKs &= p_{18}\cdot y_{K_{ECF}}\cdot y_{DMI}\\
KeKt &= H^{+}(y_{K_{GIT}},p_{15};10)\cdot p_{25}\cdot y_{K_{ECF}}\cdot H^{-}(y_{K_{TISS}}-p_{10},p_{2}-p_{10};10)\\
KeKu &= (1+p_{13}\cdot H^{+}(y_{K_{ECF}},p_{24};5))\cdot p_{6}\cdot y_{K_{GIT}}\cdot (1+p_{16}\cdot H^{+}(y_{K_{ECF}},p_{22};10))+p_{53}\cdot H^{+}(y_{K_{ECF}},p_{89};2)\\
KeMi &= p_{52}\cdot p_{55}\cdot H^{+}(y_{K_{ECF}},p_{40};10)\\
KeR &= p_{4}\cdot y_{K_{ECF}}\\
KeKi &= (p_{8}+p_{9}\cdot H^{+}(y_{Ins},p_{3};8))\cdot H^{+}(y_{K_{ECF}},p_{33};2)\cdot (1+p_{21}\cdot H^{+}(y_{pH},p_{20};10))\\
KfKg &= p_{30}\cdot y_{K_{FEED}}\\
KgKe &= p_{31}\cdot y_{K_{GIT}}\\
KiKe &= (1+H^{-}(y_{K_{ECF}},p_{29};10))\cdot p_{5}\cdot (1+H^{-}(y_{pH},p_{20};10))\cdot H^{+}(y_{K_{ICF}},p_{23};2)\cdot (1+p_{19}\cdot H^{+}(y_{K_{ICF}},p_{14};2))\\
KsKg &= p_{32}\cdot y_{K_{SAL}}\\
KtKe &= H^{-}(y_{K_{GIT}},p_{15};10)\cdot p_{26}\cdot (p_{27}-y_{K_{ECF}})\cdot H^{+}(y_{K_{TISS}}-p_{10},p_{38}\cdot 0.99-p_{10};10)\\
SnkGb &= H^{+}(y_{Gluc_{B}},p_{58};10)\cdot (p_{28}\cdot y_{Gluc_{B}}+p_{59}\cdot y_{Gluc_{PROD}}\cdot e^{-p_{66}\cdot p_{55}}+p_{55}\cdot p_{47})
\end{aligned}$$

Model parameters

Parameters displayed here are identical to the values used in the SBML Code available in the BioModels Database. Note that some parameter indices are missing. The total number of parameters is 64.

Par.	Value	Unit	Explanation
p_1	40.4	$\frac{g}{h}$	Hill threshold for $Gluc_{FEED}$ in the rate $GsGb$
p_2	1600	g	Hill threshold for K_{TISS}
p_3	22	$\frac{\mu U}{ml}$	Hill threshold for $Insulin$
p_4	0.0001783	$\frac{L}{h}$	Clearance rate for K_{ECF}
p_5	0.563	$\frac{g}{h}$	Basic shift rate of potassium in $KiKe$
p_6	0.051	$\frac{1}{h}$	Scaling factor for the rate $KeKu$
p_7	0.925	$\frac{g}{L}$	Hill threshold for glucose in the rate $GsGb$
p_8	0.5994	$\frac{g}{h}$	Basic shift rate of potassium in $KeKi$
p_9	0.4016	$\frac{g}{h}$	Scaling factor for Hill function in the rate $KeKi$
p_{10}	1400	g	Adjustment of threshold in the Hill function for K_{TISS}
p_{12}	0.0105	$\frac{L \cdot mL}{g \cdot h \cdot \mu U}$	Scaling factor for the rate $GsGb$
p_{13}	6.08	-	Scaling factor for Hill function in the rate $KeKu$
p_{14}	0.15639932	$\frac{g}{L}$	Hill threshold for K_{ICF}
p_{15}	32	g	Hill threshold for K_{GIT}
p_{16}	10	-	Scaling factor for Hill function in the rate $KeKu$
p_{17}	16	$\frac{L}{h}$	Basic rate of glucose in the rate $GsGb$
p_{18}	0.0223	$\frac{L}{g}$	Basic rate of potassium in the rate $KeKs$
p_{19}	0.1373	-	Scaling factor for Hill function in the rate $KiKe$
p_{20}	7.38	-	Hill threshold for pH
p_{21}	0.1085	-	Scaling factor for Hill function in the rate $KeKi$
p_{22}	0.25	$\frac{g}{L}$	Hill threshold for K_{ECF}
p_{23}	0.58649745	$\frac{g}{L}$	Hill threshold for K_{ICF}
p_{24}	0.1962811466	$\frac{g}{L}$	Hill threshold for K_{ECF}
p_{25}	6.6085	$\frac{L}{h}$	Scaling factor for the rate $KeKt$
p_{26}	49.0279	$\frac{L}{h}$	Scaling factor for the rate $KtKe$
p_{27}	0.232634885	$\frac{g}{L}$	Threshold for K_{ECF}
p_{28}	25	$\frac{L}{h}$	Fraction of $Gluc_B$ for body use
p_{29}	0.11729949	$\frac{g}{L}$	Hill threshold for K_{ECF}
p_{30}	0.95	-	Fraction of K_{FEED} passing to K_{GIT}
p_{31}	0.353	$\frac{1}{h}$	Fraction of K_{GIT} absorbed to K_{ECF}
p_{32}	1.31	$\frac{1}{h}$	Fraction of K_{SAL} passing to K_{GIT}
p_{33}	0.07819966	$\frac{g}{L}$	Hill threshold for K_{ECF}
p_{35}	100	g	Hill threshold for $Gluc_{STORE}$
p_{36}	1	$\frac{L}{h}$	Hill threshold for milk production
p_{38}	1509.6	g	Hill threshold for K_{TISS}
p_{39}	30	$\frac{1}{h}$	Basic rate of glucose in the rate $GpGb$
p_{40}	0.01	$\frac{g}{L}$	Hill threshold for K_{ECF}
p_{41}	20	$\frac{L}{h}$	Factor for insulin clearance
p_{42}	4.5	$\frac{1}{h}$	Factor for DMI-depentend stimulation of metabolic activity
p_{43}	10	$\frac{g}{h}$	Hill threshold for DMI
p_{44}	1.2	$\frac{1}{h}$	Decay rate of metabolic activity

Table S1. Parameter values.

Par.	Value	Unit	Explanation
p_{45}	3.0	$\frac{1}{h}$	Basic stimulation of metabolic activity
p_{46}	0.3	-	Fraction of glucose and glucogenic substances in DMI
p_{47}	72.0	$\frac{g}{L}$	Glucose for milk production
p_{48}	0.08	-	Glucose directly absorbed from $Gluc_{FEED}$ to $Gluc_B$
p_{49}	800	$\frac{\mu U \cdot L}{mg \cdot h}$	Scaling factor for $Gluc_B$ influencing insulin production
p_{50}	0.4	$\frac{g}{L}$	Hill threshold for $Gluc_B$
p_{51}	0.1	$\frac{1}{h}$	Basic rate of glucose in the rate $GpGs$
p_{52}	1.4	$\frac{g}{L}$	Scaling factor for the rate $KeKm$
p_{53}	0.25	$\frac{g}{h}$	Basic part of rate $KeKu$
p_{54}	1.0	$\frac{g}{h}$	Factor for variation of feed intake
p_{55}	0.0	$\frac{L}{h}$	Milk production
p_{56}	0.01137	-	Potassium content in DMI
p_{57}	3800	g	Hill threshold for $Gluc_{STORE}$
p_{58}	0.01	$\frac{g}{L}$	Hill threshold for $Gluc_B$
p_{59}	1.0	$\frac{1}{h}$	Fraction of $Gluc_{PROD}$ for body use
p_{60}	2.0	$\frac{g}{h}$	Basic rate of glucose in the rate $GsGp$
p_{61}	5.0	$\frac{g}{h}$	Hill threshold for $Gluc_{FEED}$
p_{62}	1.0	$\frac{L}{h}$	Hill threshold for milk production
p_{63}	800	$\frac{g}{h}$	Scaling factor for the rate $GsGb$ during milk production
p_{64}	0.2	$\frac{L}{h}$	Hill threshold for milk production
p_{65}	0.3	$\frac{g}{L}$	Hill threshold for $Gluc_B$
p_{66}	1.4	$\frac{h}{L}$	Scaling factor for reduction of glucose body use during milk production
p_{89}	0.001	$\frac{g}{L}$	Hill threshold for K_{ECF}

Table S2. Parameter values continued.
